# Clinical investigation of glucokinase activators for the restoration of glucose homeostasis in diabetes

**DOI:** 10.1111/1753-0407.13544

**Published:** 2024-04-25

**Authors:** Ping Li, Dalong Zhu

**Affiliations:** ^1^ Department of Endocrinology Drum Tower Hospital Affiliated to Nanjing University Medical School Nanjing China

**Keywords:** dorzagliatin, glucokinase, glucose homeostasis, type 2 diabetes

## Abstract

As a sensor, glucokinase (GK) controls glucose homeostasis, which progressively declines in patients with diabetes. GK maintains the equilibrium of glucose levels and regulates the homeostatic system set points. Endocrine and hepatic cells can both respond to glucose cooperatively when GK is activated. GK has been under study as a therapeutic target for decades due to the possibility that cellular GK expression and function can be recovered, hence restoring glucose homeostasis in patients with type 2 diabetes. Five therapeutic compounds targeting GK are being investigated globally at the moment. They all have distinctive molecular structures and have been clinically shown to have strong antihyperglycemia effects. The mechanics, classification, and clinical development of GK activators are illustrated in this review. With the recent approval and marketing of the first GK activator (GKA), dorzagliatin, GKA's critical role in treating glucose homeostasis disorder and its long‐term benefits in diabetes will eventually become clear.

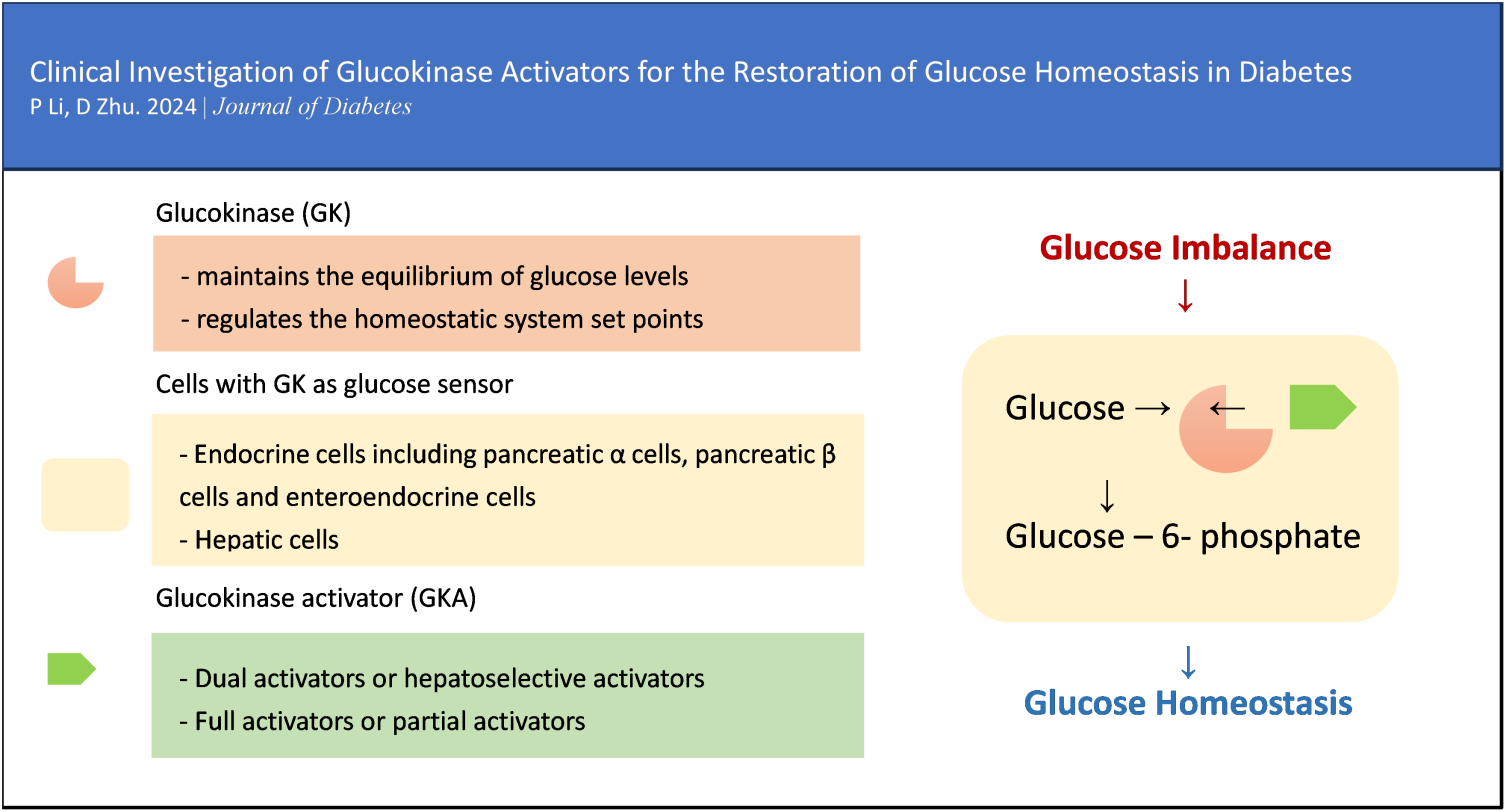

## Graphical abstract

Highlights

- The crucial role of glucokinase (GK) in the homeostatic system maintains the balance of glucose levels.
- GK also influences the secretion of essential endocrine hormones like glucagon and glucagon‐like peptide‐1 (GLP‐1).
- Studies in a broader population of patients with diabetes and prediabetes will provide greater clinical evidence for the long‐term effects of GK activation.

## 1 INTRODUCTION

Diabetes, a metabolic disorder characterized by hyperglycemia, is a prevalent disease caused by a progressive decline in glucose homeostasis. In theory, this glucose imbalance can be corrected by a homeostatic control system.^1^ However, in patients with diabetes, the system is constantly challenged by two primary factors: defective insulin secretion by pancreatic β cells and the incapacity of insulin‐sensitive tissues to respond to insulin.^2^ In addition, long‐term disruption of glucose homeostasis dramatically increases the risk of endothelial dysfunction, which can initiate cardiovascular disease and diabetic kidney disease.^3^

Glucokinase (GK) is a unique kinase (hexokinase‐IV) in mammals that acts as a sensor to regulate glucose homeostasis.^4^ It governs the set points of the homeostatic system, which has a significant impact on glucose‐stimulated insulin secretion (GSIS).^5^, ^6^ GK also influences the release of numerous important endocrine hormones, including glucagon and glucagon‐like peptide‐1 (GLP‐1).^7^, ^8^, ^9^, ^10^ The net result of numerous hormone competition or intergradation inside pancreatic islets or the gastrointestinal tract can maintain the homeostatic system. GK's essential involvement in the homeostatic system keeps glucose levels in balance.^11^

Unfortunately, GK expression and activity in the pancreas and liver were significantly decreased in patients with type 2 diabetes (T2D).^12^, ^13^ On the other hand, a recent report has suggested that T2D patients with better glycemic control have higher levels of β‐cell GK expression.^14^ The aforementioned data have provided a framework for investigating the potential therapeutic justification for targeting GK in patients with diabetes to reestablish their glucose homeostasis. Recently published reviews have provided us with comprehensive overviews and structural insights regarding the development of therapeutic candidates.^15^, ^16^ The objective of this review is to present the current clinical investigation aimed at restoring glucose homeostasis in diabetes, with a specific emphasis on the GK target.

## 2 GLUCOKINASE ACTIVATION

**FIGURE 1:**
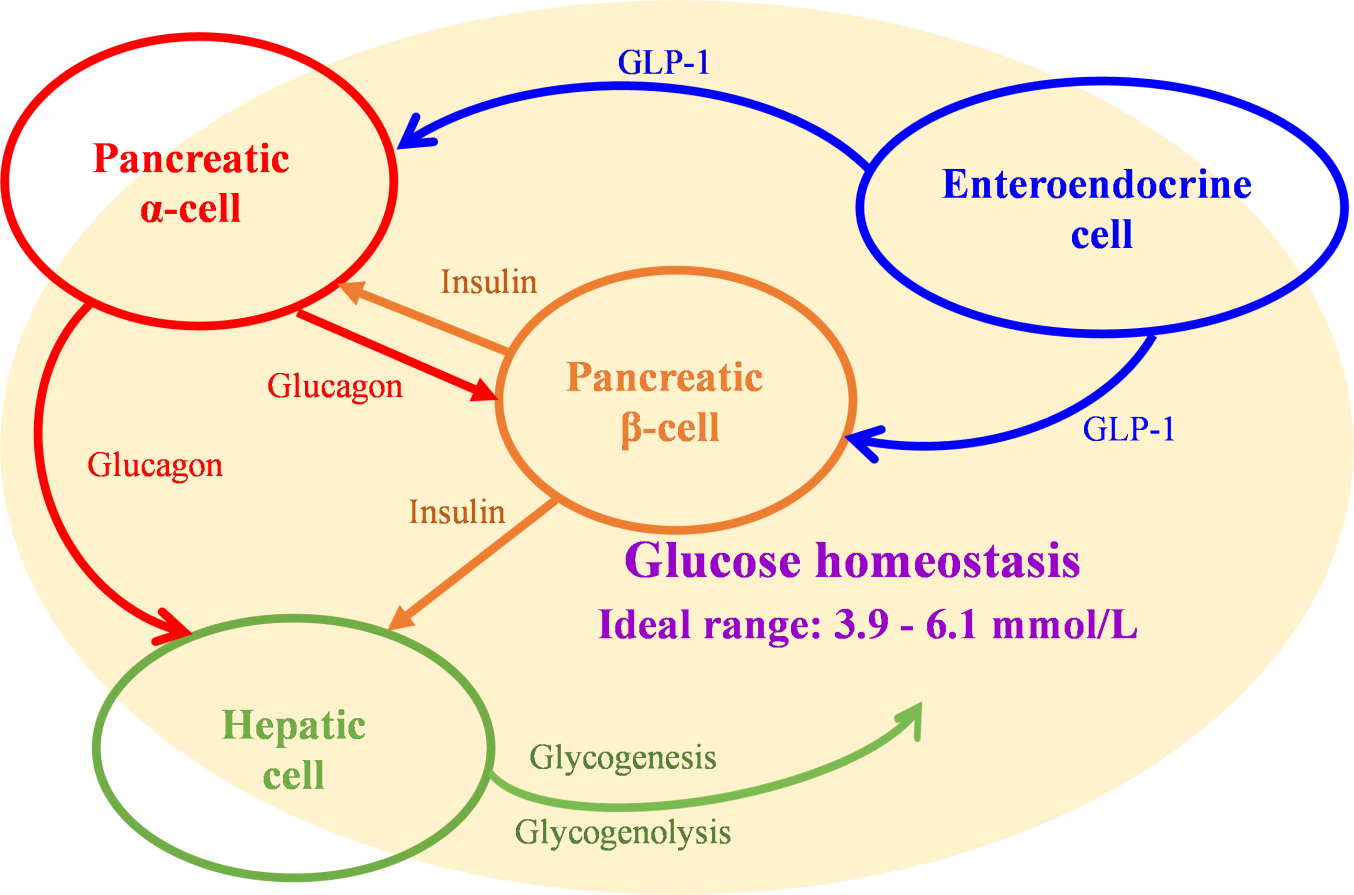
Glucose homeostasis modulated by cells with GK as a glucose sensor. GK, glucokinase; GLP‐1, glucagon‐like peptide‐1.

Due to its essential role in maintaining whole‐body glucose homeostasis (Figure 1), GK has a unique ability to self‐regulate its activity via slow conformational dynamics. This allows for a cooperative response to glucose, which is important to stabilize glucose levels.^17^ GK reaches half‐maximal activity at a glucose concentration of 8.0 mmol/L, whereas the other three hexokinases become saturated at substantially lower blood glucose values (less than 1.0 mmol/L). Consequently, the glucose metabolism regulated by GK increases as blood glucose levels rise from fasting to postprandial levels following the consumption of a meal abundant in carbohydrates.^18^, ^19^ GK exhibits a non‐Michaelis–Menten sigmoidal dependence on glucose concentration. The inflection point of the curve lies between 4 and 5 mmol/L, which is comparable to the insulin secretion threshold. This results in a guaranteed graded response to changes in glucose levels. When glucose levels approach the physiological limit for glucose‐induced insulin secretion, GK activity reaches a plateau.^20^, ^21^

### 2.1 In endocrine cells

GK activation in pancreatic β‐cells is a rate‐limiting step in glucose phosphorylation that elicits glucose‐stimulated insulin secretion to maintain glucose homeostasis.^22^ This was demonstrated in a 28‐day treatment clinical study, where GK activation resulted in significant improvement of β‐cell function and enhancement of glycemic control.^23^

Although GK activation is primarily associated with β‐cells, it is also expressed in multiple endocrine cells, including enteroendocrine cells, pancreatic α‐cells, pancreatic δ‐cells, and cells in the anterior pituitary.^4^ Modulation of GK activity by pharmacological activators and inhibitors has been shown to lower or raise the glucose threshold for glucagon release from single α‐cells.^24^, ^25^ GK is also required for glucose‐dependent regulation of GLP‐1 secretion. In a phase 1 open‐label clinical trial, glucose homeostasis in patients with T2D was observed to be regulated not only by GK activation in the pancreas and liver but also through the improvement of GLP‐1 release.^26^

### 2.2 In hepatic cell

In humans, when blood glucose levels are below about 10 mmol/L, hepatic GK remains inactive by forming a complex with an endogenous inhibitor called the glucokinase regulatory protein (GKRP). This results in an even lower affinity for glucose in hepatic cells than in pancreatic β‐cells. Generally, hepatic GK is activated only after meals to carry out its role of boosting hepatic glucose uptake.^27^, ^28^ As a competitive inhibitor for glucose, GKRP locks GK within the nucleus, and activated GK encourages hepatic glucose absorption as well as glycogenesis and storage.^29^, ^30^ In individuals with impaired fasting glucose, low‐dose fructose experimentally enhanced hepatic GK activity and restored hepatic glucose sensing.^31^ In a clinical trial with 6 months of continuous treatment, hepatoselective GK activation might considerably lower fasting glucagon concentrations in patients with T2D.^32^ It implies that a single GK activation in the liver may affect pancreatic hormone secretion through a bypass mechanism.

## 3 CLINICAL EXPLORATION ON GK TARGET

Since RO0281675 became the first glucokinase activator (GKA) in research and development, GKAs and other novel therapeutic pharmaceuticals have been investigated clinically for decades.^33^, ^34^

GKAs can be divided into dual activators and hepatoselective activators based on the specificity of their GK targets. Dual activators are pharmaceuticals that can activate GK in multiple organs, including the pancreas and gastric intestine, in addition to the liver. Dual activators could influence glucose homeostasis via the pancreas, which would support long‐term therapy. Hepatoselective activators prevent drug molecules from accessing the pancreas and prevent GSIS from elevating GK expression in the liver, thereby reducing the incidence rate of hypoglycemia.^35^

By their effect on the kinetic parameters of the allosteric enzyme, GKAs can be categorized as either full or partial activators. Full activators can attain a desirable fasting/postprandial glucose level due to their high efficiency and rapid onset of action. With chemical designs aimed at preventing excessively low glucose S_0.5_ levels,^36^ partial activators typically have moderate efficacy and onset speed. Their therapeutic windows are considerably larger.

**FIGURE 2:**
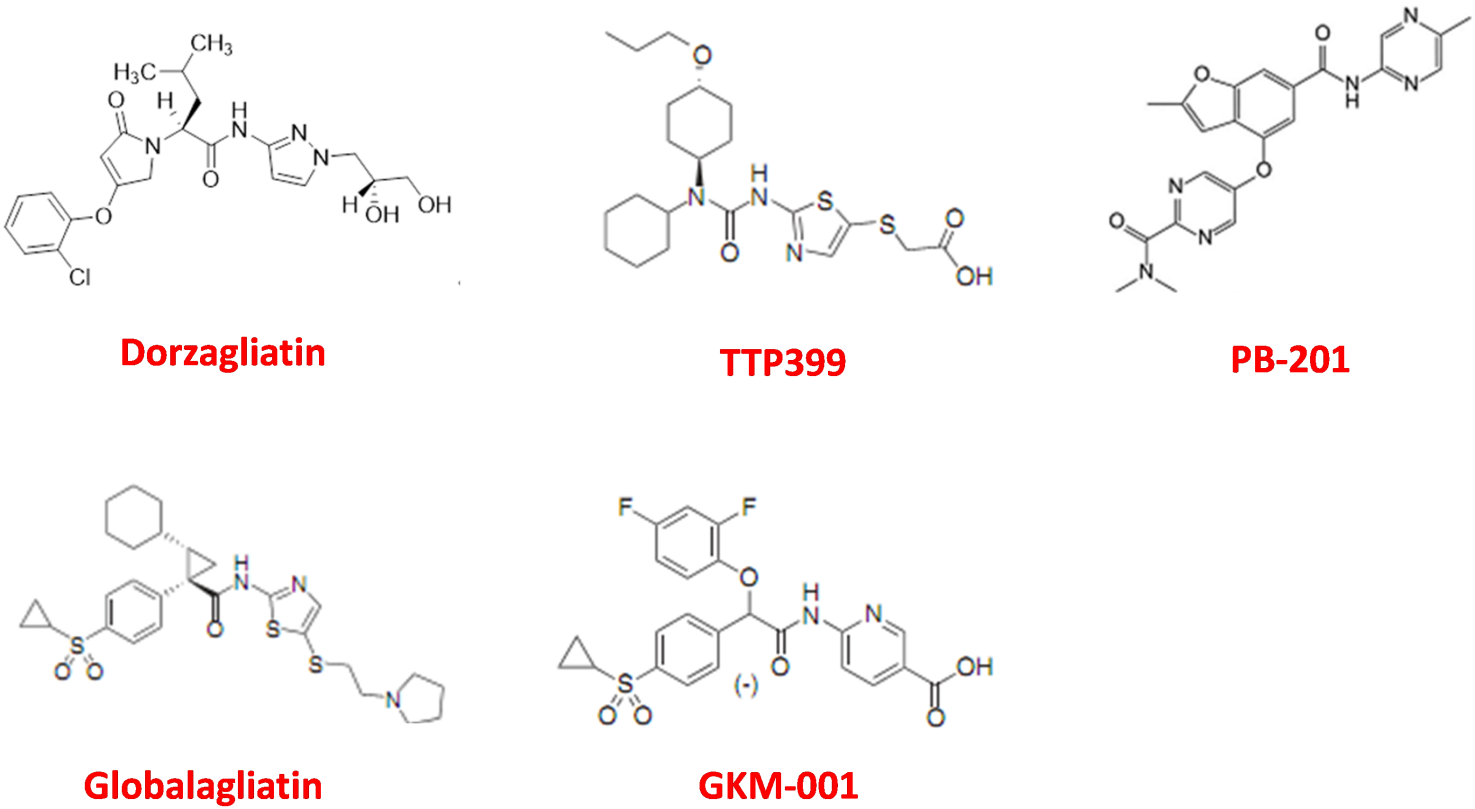
Structure of GK activators. GK, glucokinase.

**FIGURE 3:**
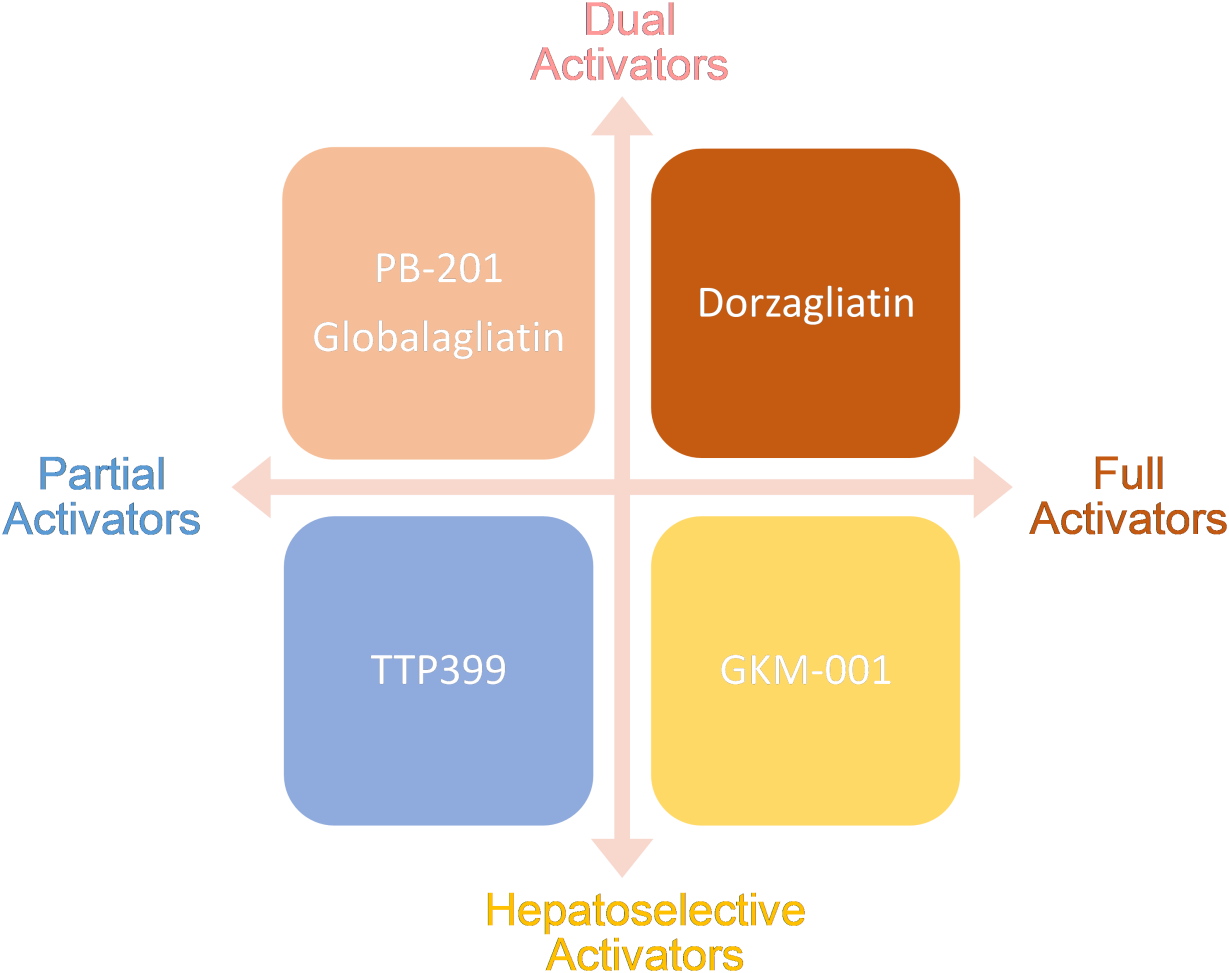
Classification of GK activators for antidiabetic treatment. GK, glucokinase.

Although there is a big difference in the designs of GKAs (Figures 2 and 3), it is important to pay close attention to the status of glucose homeostasis in the treatment by GKAs. If one type of GKA is not effective in improving glucose homeostasis in patients with T2D, tachyphylaxis and adverse events in the metabolic system will be observed in clinical practice. The initial failure of MK‐0941 in phase 2 clinical trials was due to clinical hypoglycemia resulting from the disturbance of the GSIS threshold, which is comparable to the levels observed when activating GK mutations are present.^37^, ^38^, ^39^

Currently, there are five synthetic drug molecules of GK targets being investigated globally. Modifying the kinetic parameters of GK can affect its glucose‐dependent activity, Therefore, only those molecules with appropriate influence on kinetic parameters of multiple GK targets can achieve the desirable profiles for the development of diabetes therapeutics.

This article presents the progress of their clinical explorations (Table 1). In addition, some plants and their phytoconstituents have been found to possess antidiabetic potential by activating the GK. However, although these plants have shown potential for increasing GK activity in animal studies, there are currently no clinical data available to demonstrate the same effects in humans.^40^

**TABLE 1:** Overview of GK activators in clinical studies.

	Dorzagliatin	TTP399	PB‐201	Globalagliatin	GKM‐001
Dosage	75 mg twice daily	800 mg once daily	100 mg twice daily	80 mg once daily	Data undisclosed
HbA1c (%)	1.0 reduction after 24w treatment	0.5 reduction after 6‐month treatment^32^	0.6 and 0.8 reduction after 12w treatment^a^	Data undisclosed	Data undisclosed
FPG (mmol/L)	0.4 reduction compared with placebo^45^	0.3 reduction compared with placebo^50^	−0.5 and 0.8 reduction compared with placebo^a^	Data undisclosed	Data undisclosed
PPG (mmol/L)	2.5 reduction compared with placebo^45^	Data undisclosed	Data undisclosed	Data undisclosed	Data undisclosed
Clinical trials registered^b^	17 trials have been registered in the name of dorzagliatin or HMS5552	3 trials have been registered in the name of TTP399 and GK1‐399	12 trials have been registered in the name of PF04937319 and PB‐201	8 trials have been registered in the name of LY2608204, globalagliatin, and SY‐004	2 trials have been registered in the name of GKM‐001
Clinical trials completed^b^	All 17 trials registered have been completed	3 trials registered have been completed	10 trials have been completed and 2 pivotal trials are in recruitment	7 trials have been completed and 1 trial has been withdrawn	2 trials registered have been completed
POC trials^b^	A 12‐week treatment study has been completed^46^	A 6‐month treatment trial in patients with T2D^50^ and a 12‐week treatment trial in patients with T1D^51^ have been completed	Two 12‐week metformin add‐on trials compared to sitagliptin and titrated glimepiride^54^	A 16‐week treatment, placebo‐controlled trial has been completed^59^	Will initiate a 12‐week phase IIb (effect on HbA1c) study
Pivotal trials^b^	Two 52‐week treatment studies have been completed^44^, ^45^, ^62^	Will initiate a phase 3 trial in patients with T1D	Two 52‐week treatment studies have been initiated	No pivotal trial has been initiated	No pivotal trial has been initiated
Effects on hormones in homeostasis	Improve early phase secretion of insulin^23^ and GLP‐1 enhancement^25^	Glucagon reduction^32^	Data undisclosed	Data undisclosed	Data undisclosed

Abbreviations: FPG, fasting plasma glucose; GK, glucokinase; GLP‐1, glucagon‐like peptide‐1; HbA1c, glycated hemoglobin; POC, proof‐of‐concept; PPG, postprandial plasma glucose; T1D, type 1 diabetes; T2D, type 2 diabetes.

^a^ PB‐201 has two POC metformin combination trials with a dosage of 100 mg once daily.

^b^ The data cut off by November 2023.

### 3.1 Dorzagliatin

Dorzagliatin (HMS5552) is a global first‐in‐class antidiabetic medication approved in China for the treatment of T2D. As an allosteric and dual‐acting full GKA, dorzagliatin features a fourth‐generation small‐molecule design.^41^ With successful commercialization, dorzagliatin is a novel antidiabetic medicine either alone or in combination with metformin, as proven by the considerable reduction in glycolated hemoglobin (HbA1c), fasting glucose, 2‐h postprandial glucose, and larger percentage of patients achieving HbA1c <7% as compared to placebo.^42^, ^43^ The 52‐week study with dorzagliatin has proven strong glycemic maintenance in the 28‐week open‐label period.^44^, ^45^ More than that, dorzagliatin offers significant proof of the improvement in glucose homeostasis. In every stage of clinical trials from phase 1 to 3, dorzagliatin has been demonstrated to increase early‐phase insulin secretion and β‐cell function and to decrease insulin resistance as assessed by routinely used indices.^23^, ^44^, ^45^, ^46^ They were maintained for 52 weeks after dorzagliatin discontinuation, suggesting sustained remission in patients with T2D.^47^ Dorzagliatin as monotherapy and as an add‐on to metformin was well tolerated with a good safety profile in patients with T2D in clinical trials.^23^, ^44^, ^45^

On the basis of GK activating effects in both pancreatic islet cells and enteroendocrine cells,^23^, ^26^ it was anticipated that dorzagliatin would play a larger role in correcting glucose homeostasis disorder. The recommended dosage of dorzagliatin is one 75 mg oral tablet twice daily, 1 h before breakfast and dinner. No dosage modification is necessary for patients with T2D and chronic kidney disease.^48^, ^49^

### 3.2 TTP399

In 2016, the first proof‐of‐concept (POC) trial for TTP399 (GK1‐399) was completed. In this randomized, double‐blind, placebo‐ and active‐controlled (sitagliptin 100 mg QD) phase 2b 6‐month treatment study, TTP399 (800 mg per day) was associated with a clinically significant and sustained decrease in HbA1c in comparison to placebo. As an oral hepatoselective GKA initially discovered for potential unmet medical needs of patients with T2D, TTP399 does not activate GK in pancreatic β‐cells, and there were no changes in fasting active GLP‐1, lactate, insulin, or C‐peptide levels during the trial. In the maximum dose group (800 mg daily), fasting glucagon concentrations decreased significantly.^50^ As TTP399 does not affect GK‐GKRP interaction in either euglycemia or hyperglycemia, the effect of TTP399 on glucose homeostasis may rely primarily on hepatic GK activation; however, the mechanism requires further investigation.^32^ The proportions of individuals reporting treatment‐emergent adverse events were comparable between groups, and no serious adverse events were reported.

The indication for TTP399 in clinical development has changed to type 1 diabetes (T1D) beginning in 2019. A phase 1b/2 adaptive study with a 12‐week treatment duration demonstrated that hepatic GK activation could simultaneously decrease HbA1c, plasma β‐hydroxybutyrate, urinary ketones, and the frequency of severe or symptomatic hypoglycemia relative to placebo.^51^, ^52^

### 3.3 PB‐201

PB‐201 (PF04937319) is a partial, pancreas‐ and liver‐dual GKA that has glucose‐dependent, moderate binding affinity and fast dissociation. It is the first partial GKA worldwide to reach the phase 3 pivotal study stage. Two phase 3 trials have been designed and started recruiting separately in 2021 and 2022. The primary objectives of these studies are to monitor the change in HbA1c with PB‐201 for patients with T2D with poor glycemic control via metformin hydrochloride monotherapy (study 302) or treatment‐naive patients with T2D (study 303) from baseline to 24 weeks in comparison with placebo (study 302) or vildagliptin and placebo (study 303). The main secondary objectives of both studies are to assess the efficacy and safety of PB‐201 following treatment for 52 weeks. It is worth noting that in phase 3 trials, homeostatic indices such as homeostatic model assessment of β‐cell function (HOMA‐β) and homeostatic model assessment of insulin resistance (HOMA‐IR) have been listed in the protocol as secondary end points.^53^

Before phase 3 trials, several POC trials demonstrated the ideal dosage of this pharmaceutical molecule. In two phase 2 trials with 12‐week treatment, the maximal dose (100 mg once daily) demonstrated an HbA1c‐lowering effect similar to that with sitagliptin and less than that with glimepiride, as well as good tolerance.^54^ Another phase 1 trial explored PB‐201's safety, tolerability, pharmacokinetics, and pharmacodynamics in the optimal dose (100 mg twice daily) with the technology of continuous glucose monitoring (CGM). The 24‐h CGM data in drug‐naïve Chinese patients with T2D provided convincing evidence that the risk of hypoglycemia and other side effects reported with previous GKAs were low with PB‐201.^55^

### 3.4 Globalagliatin

Oral dual GKA globalagliatin (LY2608204, SY‐004) targets both pancreatic β‐cells and hepatocytes. In five completed phase 1 studies, including two in Chinese subjects with 28‐day dose‐escalating treatments, 2–120 mg and 20‐320 mg globalagliatin demonstrated positive tolerability and safety for healthy subjects and patients with T2D, respectively, with an evident antihyperglycemic effect.^56^, ^57^ Analyzing homeostatic indices such as HOMA‐β and HOMA‐IR, globalagliatin decreased HOMA‐IR in the high‐dose group.^57^ Two phase 2 POC trials of the candidate have been completed at this point. The first is a 12‐week, phase 2, randomized, double‐blind, active‐controlled (glimepiride) study of LY2608204 as monotherapy or in combination with metformin in patients with T2D.^58^ The second is a placebo‐controlled, 16‐week treatment trial that was completed in 2021 to activate the pivotal trial. The phase 2 trial assessed the efficacy and tolerability of various doses of SY‐004 in patients with T2D.^59^

### 3.5 GKM‐001

Unlike dual activators, GKM‐001 appears not to affect insulin secretion as evidenced by the lack of increase in C‐peptide in patients with T2D. With the aid of a hyperglycemic clamp, it has been confirmed to act selectively on hepatic GK. In a multiple ascending dose trial with dosage panels from 25 mg to 1000 mg BID, a 9% to 20% lowering of 24 h‐plasma‐glucose had been observed in patients with T2D. With 12 h of overnight and 2 h of the fasting period, it demonstrated no hypoglycemia at a steady state.^60^ A predicted change based on the 14‐day treatment with GKM‐001 shows a dose‐dependent reduction in HbA1c. At this moment further evidence is still required on GKM‐001 to complete dose selection for the pivotal trial.

## 4 PERSPECTIVE

GK's core position in the modulation of glucose homeostasis provides a good drug target to evaluate the importance of glucose control in daily health care. From the clinical studies conducted on GKAs so far, we can find that monitoring biomarkers of glucose homeostasis is usually defined as secondary endpoints. A series of clinical trials of dorzagliatin, the globally first approved GKA, systematically evaluated the impact of GK activation on blood glucose homeostasis. TTP399, as a hepatoselective GKA, showed the characteristics of reducing fasting glucagon of T2D.^50^ In the early phase studies of PB‐201 and GKM‐001, CGM technology has been used to detect time in range, time beyond range, time above range, and 24 h‐plasma‐glucose after treatment, respectively.^55^, ^60^

The second point is that, unlike some antidiabetic drugs such as SGLT‐2 inhibitors or GLP‐1 receptor agonists, which have been demonstrated to have special mechanisms to improve cardiovascular outcomes, GK activators build their efficacy directly on the recovery of GK target to achieve ideal glucose control, which may have stronger protective effects on coronary artery disease and heart failure than nontargeted glucose‐lowering regimens.^61^ Because impaired glucose homeostasis occurs at a very early stage of diabetes, studies with a larger population of patients with diabetes and prediabetes will add more clinical evidence to the long‐term benefits of GK activation.

**FIGURE 4:**
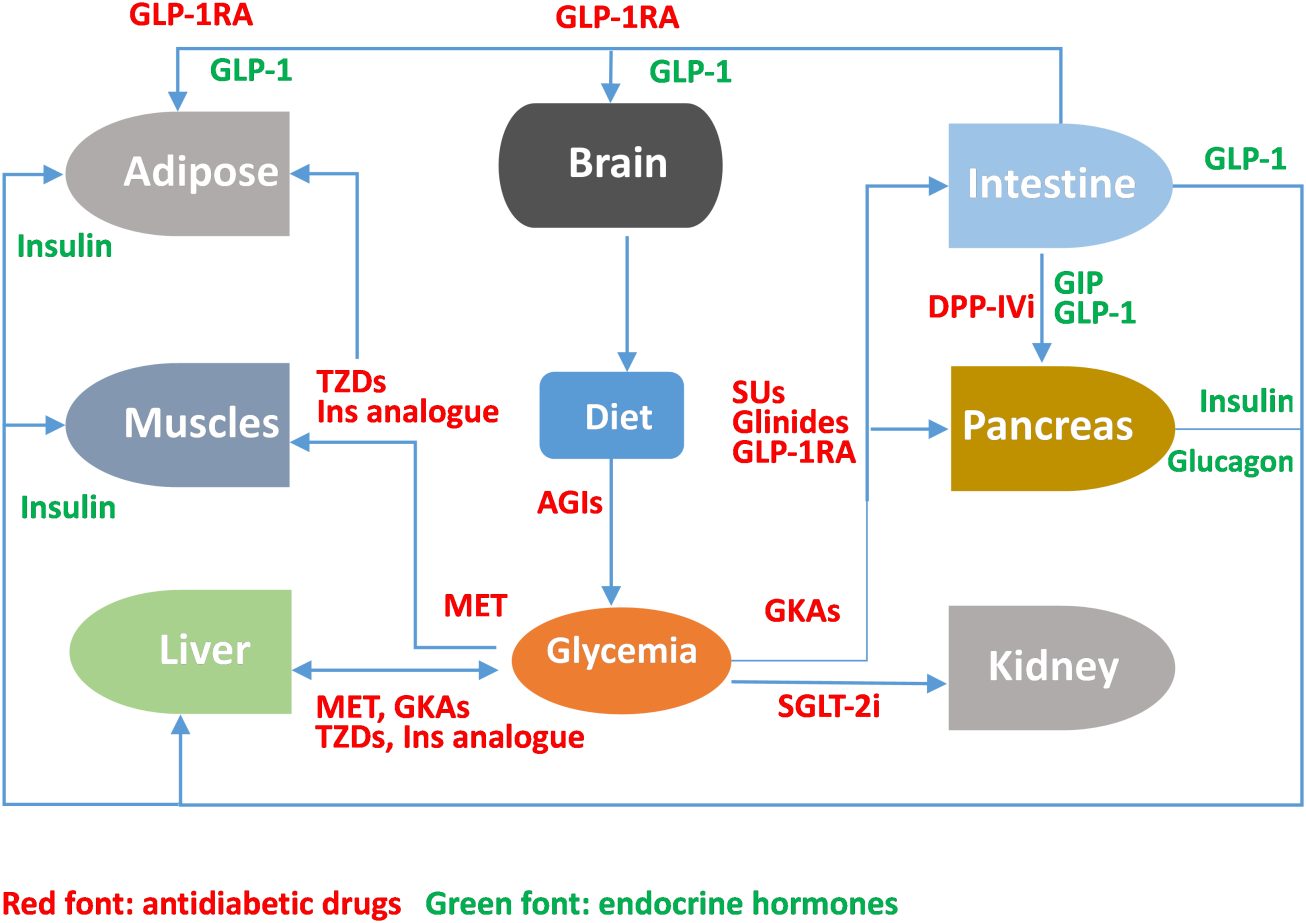
Mechanism of antidiabetic drugs. AGI, alpha‐glucosidase inhibitor; DPP‐IVi, dipeptidyl peptidase IV inhibitor; GIP, gastric inhibitory polypeptide; GKA, glucokinase activator; GLP‐1RA, glucagon‐like peptide‐1 receptor agonist; MET, metformin; SGLT‐2i, sodium glucose transporter 2 inhibitor; SU, sulfonylurea; TZD, thiazolidinedione.

The antidiabetic mechanism of GK regulation demonstrated that targeting the GK could be a promising approach for treating T2D and has opened new possibilities for drug discovery (Figure 4). However, the early development of certain therapeutic compounds was based on a very limited understanding of GK activation. Following the failure of several clinical trials, the field had been forced to reconsider how to use GK's crucial function in restoring glucose homeostasis. After decades of clinical investigation, a substantial body of clinical evidence has been gathered, leading to the successful commercialization of the GK activator. The future long‐term benefits for patients with T2D will become progressively clearer.

## DISCLOSURE

Zhu Dalong is an editorial board member of the *Journal of Diabetes* and a coauthor of this article. To minimize bias, they were excluded from all editorial decision‐making related to the acceptance of this article for publication.
